# Stemformatics: visualize and download curated stem cell data

**DOI:** 10.1093/nar/gky1064

**Published:** 2018-11-08

**Authors:** Jarny Choi, Chris M Pacheco, Rowland Mosbergen, Othmar Korn, Tyrone Chen, Isha Nagpal, Steve Englart, Paul W Angel, Christine A Wells

**Affiliations:** 1 Centre for Stem Cell Systems, Anatomy and Neuroscience, The University of Melbourne, Melbourne, Victoria 3010, Australia; 2 Australian Institute for Bioengineering and Nanotechnology, The University of Queensland, Brisbane, Queensland, Australia; 3 Walter and Eliza Hall Institute of Medical Research, Parkville, Victoria 3052, Australia

## Abstract

Stemformatics is an established gene expression data portal containing over 420 public gene expression datasets derived from microarray, RNA sequencing and single cell profiling technologies. Developed for the stem cell community, it has a major focus on pluripotency, tissue stem cells, and staged differentiation. Stemformatics includes curated ‘collections’ of data relevant to cell reprogramming, as well as hematopoiesis and leukaemia. Rather than simply rehosting datasets as they appear in public repositories, Stemformatics uses a stringent set of quality control metrics and its own pipelines to process handpicked datasets from raw files. This means that about 30% of datasets processed by Stemformatics fail the quality control metrics and never make it to the portal, ensuring that Stemformatics data are of high quality and have been processed in a consistent manner. Stemformatics provides easy-to-use and intuitive tools for biologists to visually explore the data, including interactive gene expression profiles, principal component analysis plots and hierarchical clusters, among others. The addition of tools that facilitate cross-dataset comparisons provides users with snapshots of gene expression in multiple cell and tissues, assisting the identification of cell-type restricted genes, or potential housekeeping genes. Stemformatics is freely available at stemformatics.org.

## INTRODUCTION

As new technologies arise, stem cells are being characterized at a far more detailed scale than ever. This data creates challenges for the stem cell research community, which include finding and navigating data that is both reliable and relevant to the biological question at hand. Stemformatics provides a curated data portal, with user friendly interfaces, for the rapid review of genes across different stem cell types, and their differentiated progeny. Anyone can request public or proprietary data to be included in the portal, and it has been set up to support cross-laboratory collaborative activities. Stemformatics was established in 2011 as part of the Stem Cells Australia initiative, and has gone on to support a global community of stem cell researchers.

**Figure 1.**
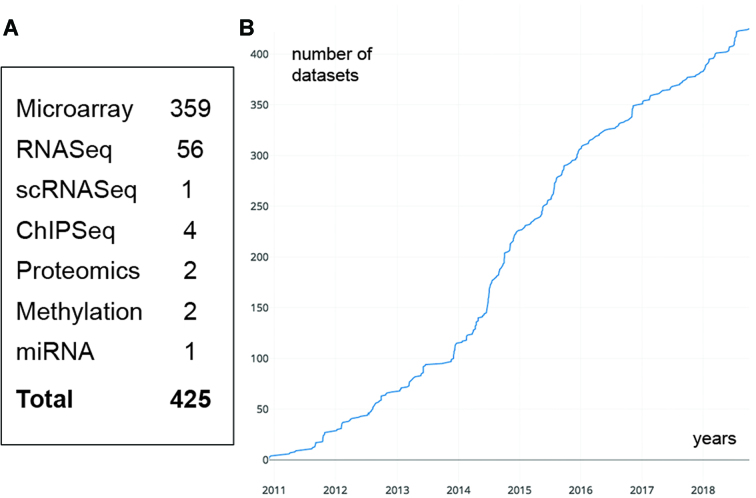
Number of public datasets in Stemformatics sorted by (**A**) platform type and (**B**) cumulative number of datasets (y-axis) curated overtime in years (x-axis).

Community focused data portals offer considerable value, providing a central resource to find relevant experimental series, and to explore the data associated with landmark publications. Stemformatics is one of a small number of stem cell portals that curate transcriptome, proteome and metabolomic data (1). Other stem cell transcriptome databases are typically limited to hosting single technological platforms. For example, both StemMapper (2) and CellFinder (3) host datasets generated on the Affymetrix microarray technology. Stemformatics is now on Version 7, which uses Ensembl human genome release 91 (hg38). It currently hosts over 600 stem cell datasets (420+ public and 180+ private datasets - note that all figures in this article have been created using public datasets only), spanning over 21 000 samples, that include a mix of technologies, with transcriptome profiling dominating the datasets included (Figure 1). Since the early days when the focus was on pluripotent stem cells, it has grown to include a broader range of stem cell types, including leukaemic cells, mesenchymal stromal cells, neural progenitors and neurons and more (Table 1). Related datasets have been placed under 'collections', such as Leukomics (https://www.stemformatics.org/leukomics) which lists datasets relevant to leukaemia research. These collections also serve as collaboration portals for communities working on large integrative analyses, such as the Project Grandiose consortium (https://www.stemformatics.org/project_grandiose) (4–6).

**Table 1.** Examples of the most common sample types hosted in Stemformatics. See details in Supplementary Data

Blood		Stem Cells/Other	
Monocyte	1600	Mesenchymal stromal cell	820
T-cell	208	Induced pluripotent stem cell	746
Macrophage	146	Acute myeloid leukaemia	553
Dendritic cell	172	Embryonic stem cell	488
Hematopoietic stem cell	199	iPSC-derived neuron	87

New additions to the site include the capacity to visualize single cell RNASeq datasets. An interactive report was specifically developed for this purpose (http://github.com/jarny/iscandar). It displays a pre-computed visualization of dimensionally reduced data, such as PCA (principal component analysis) or t-SNE (7), but is amenable to any dimensional reduction approach. The report then allows the user to visualize gene expression as a colour gradient overlaid on the cluster diagram. The report facilitates an effective data analysis cycle between the biologist and the bioinformatician, providing the means for biologists to visually explore the data while consulting on more sophisticated analyses. This embodies Stemformatics’ underlying philosophy, which aims to present the data or the results of external analysis as faithfully as possible, whilst facilitating cross-disciplinary collaboration.

**Figure 2.**
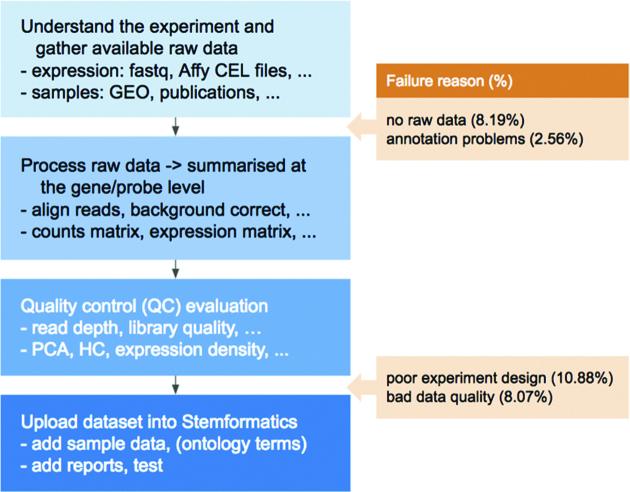
Overview of the Stemformatics pipeline from raw file stage to an online version, including common causes of data processing failure

**Figure 3.**
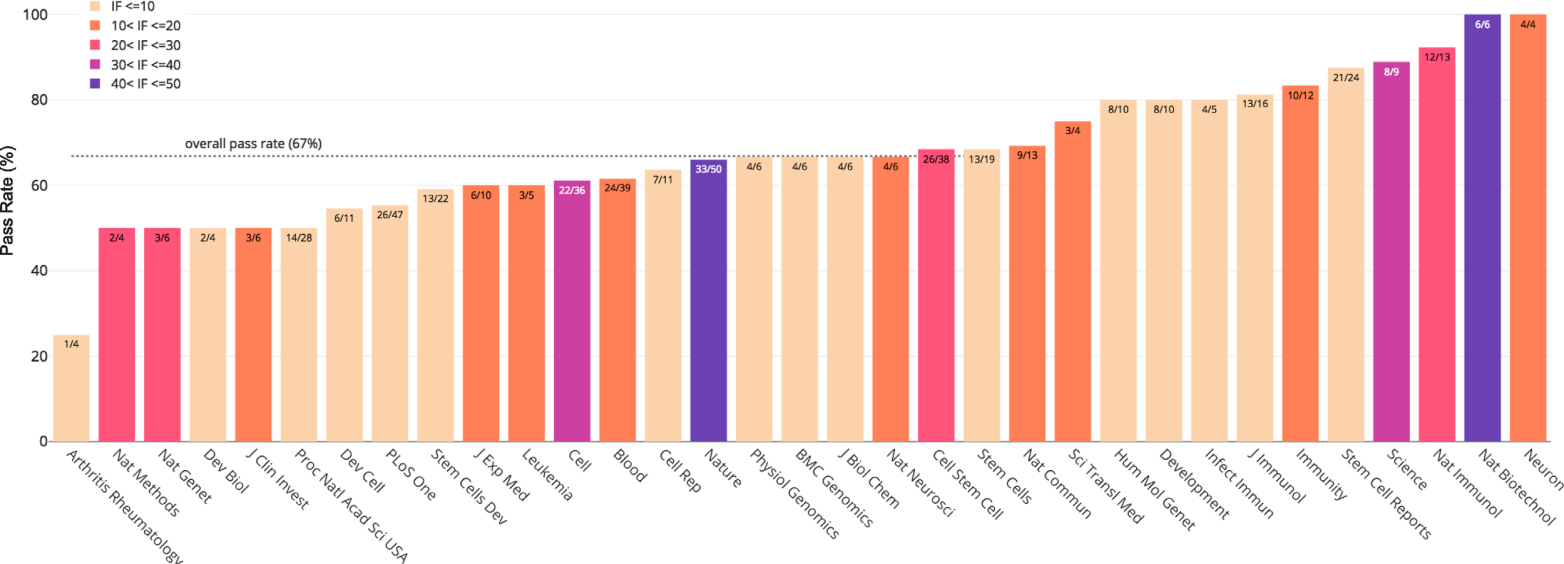
Percentage of datasets passing quality control for journal of publication (not all journals are shown). Numbers on each bar indicate the number of passed/total datasets and colours indicate the impact factor range of the journal.

A key feature of Stemformatics is that all hosted datasets have been processed from the raw data, using a set of quality control metrics (Figure 2), unlike most other large data repositories such as ArrayExpress (8) or Gene Expression Omnibus (9). An example of a data portal with its own data curation and processing pipeline is Expression Atlas (EA) (10). However Stemformatics hosts a far greater number of stem cell datasets, while EA hosts other species and includes tissue specific data. Each portal also hosts a very different set of tools. About 30% of the datasets processed by Stemformatics are rejected as a result of the stringent data processing pipeline. A common issue is poor experimental design, such as technical batch confounding biological classes of interest, but also includes lack of sample class replication, obvious misannotation of samples, or ’accession hacking’, where a portion of the data described in a publication is withheld from the public repository. This high fail rate of published datasets points to a systematic failure of peer review to evaluate the primary data accompanying many publications, regardless of the impact factor of the journal (Figure 3 and Supplementary Data).

## INTERACTIVE ANALYSIS REPORTS

The majority of data views in Stemformatics work within individual experiments. The gene search, experiment search and gene views were described in the original 2013 Stemformatics paper (11). Here, we highlight the recent additions to the site.

In addition to the interactive single cell RNASeq dataset report mentioned in the introduction, there is a Principal Components Analysis (PCA) plot provided for each dataset as an interactive viewer. This allows the user to assess distribution of samples in a dataset relative to experimental variables (sample class, experimental batch, or QC metrics). For a subset of datasets, we provide an interactive differential expression report, generated from a limma pairwise analysis and visualized via Glimma (12). Rather than simply having a static list of genes to work with, a Glimma report enables the user to interact with a table format of ranked genes, highlighting position in a volcano plot for that pairwise comparison, and across the entire data series for an experimental series.

## DATA MINING

The capacity to combine data from several experiments for additional meta-analyses is one of the opportunities that arise from a substantive collection of curated, thematic data. We see two common use cases: (i) meta-analysis that provides insight into classification or characterization of stem cell biology and (ii) a curated data for bioinformatic method development. Stemformatics aims to facilitate the bioinformatics community interested in data mining by providing a data portal where data has been transformed using uniform processing pipelines. Stemformatics provides a multi dataset download page, where a bioinformatician can obtain a script to download multiple dataset files autonomously.

**Figure 4.**
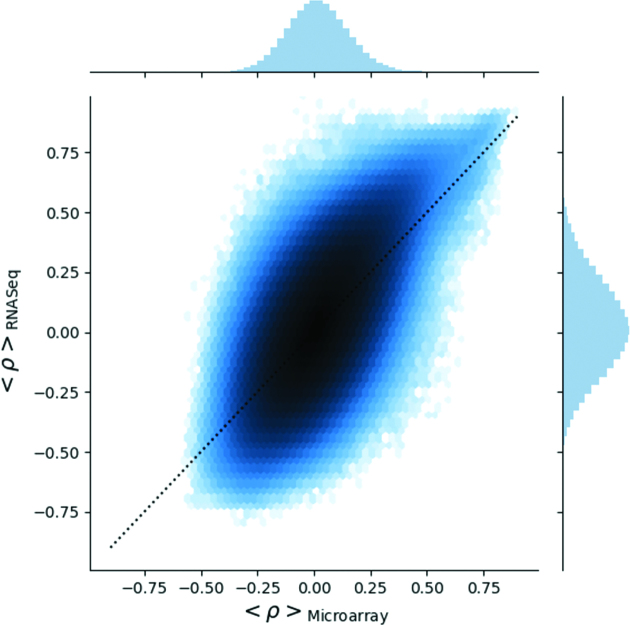
The 2D distribution of the average gene-gene pair Pearson correlation coefficient. Each point is a gene-gene pair, and its average correlation coefficient within each platform. The x-axis shows the average correlation across microarrays, and the y-axis the average across RNA Seq datasets. Colour gradient indicates areas of high point density (in log scale). Dotted black line is a 1:1 relation for reference. This plot highlights gene pairs with a consistently high correlation in both microarray and RNA Seq data in the top right hand corner.

The majority of data available for early stem cell studies, including rarer tissue stem cell subsets are restricted to microarray platforms. With the increasing trend towards RNA-seq platforms, the challenge of combining data is significant. Microarray and RNA sequencing data have significantly different distributions and linear ranges. Nevertheless, the high level of pairwise correlation shared between gene pairs measured on either platform indicates a high degree of concordance of the underlying biology, regardless of the platform used to measure it (Figure 4). Details of the calculation are available in Supplementary Data.

## INTEGRATIVE DATA ANALYSIS TOOLS

With increased numbers of datasets comes the challenge of combining data and extracting meaning from such combinations. Batch effects are well known, and derived from any number of sources (13) and there is no universally accepted method for removing these effects, so this is generally a difficult problem. As data portals become more prevalent and continue to grow, we will require more sophisticated integrative data analysis tools that can leverage the statistical power contained within the collective datasets rather than treating them as disparate containers.

**Figure 5.**
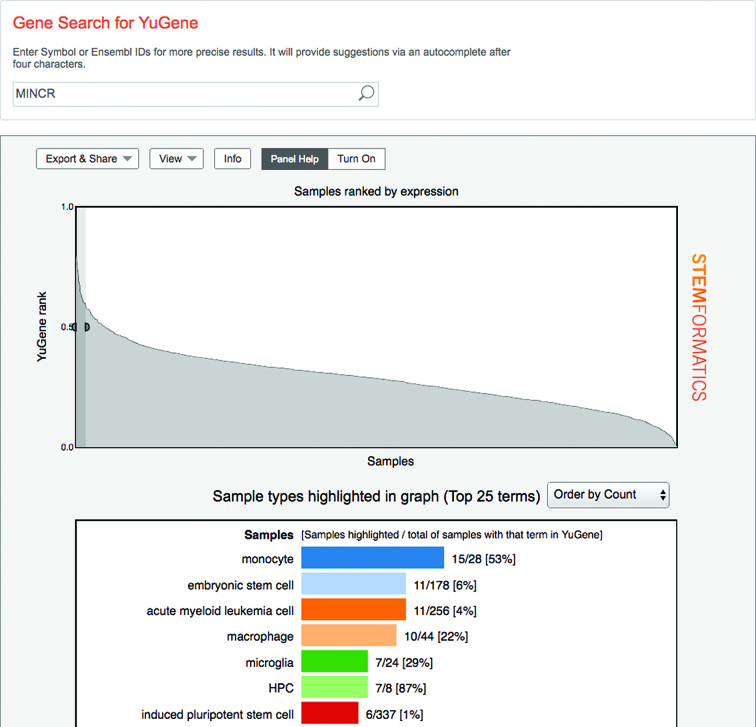
A screenshot of the Yugene graph for MINCR gene (top) with break-down of cell types for samples with high Yugene values (bottom) within the selected window.

The Yugene interactive graph, which was implemented in 2014 in Stemformatics, exemplifies our efforts towards integrative data analyses. The principal motivation was to answer the question 'Where is my gene of interest expressed across all the sample types in Stemformatics?' The graph ranks all samples across multiple applicable datasets from the highest to the lowest expression for the selected gene. A user can highlight regions of the graph, to display an interactive bar chart that shows sample biotypes that are enriched in the selected region (Figure 5).

The graph is an online implementation of the published method (14). The log expression values of all genes in a sample are converted into rank based and scaled *cumulative distribution values*, so that a Yugene value ranges from 0 (lowest rank) to 1 (highest rank). Note that currently the Yugene graph only uses microarray datasets, as the published method we are using was only applied to this data type. Due to the considerable size of the microarray datasets we have (350+), the method performs well nevertheless.

Any user can request a dataset to be uploaded into Stemformatics using an online form and a requested dataset will go into our processing queue. This means that Stemformatics also works as a collaborative platform rather than a simple container for datasets, and numerous projects have leveraged this feature, resulting in publications across a range of topics (4) (Larson *et al.* bioRxiv, doi: 10.1101/395202, 2018). Usually, unpublished data will remain private until the study is published and the time line for this is very project specific. This workflow also means that the choice of datasets to be included is driven by community demand, and collaborative project goals. Stemformatics brings together biologists, bioinformaticians and open source code developers: In 2018, Stemformatics was accepted as one of the mentor organisations for the prestigious Google Summer of Code program (https://summerofcode.withgoogle.com/), which brings together students and organisations on free open source projects.

**Figure 6.**
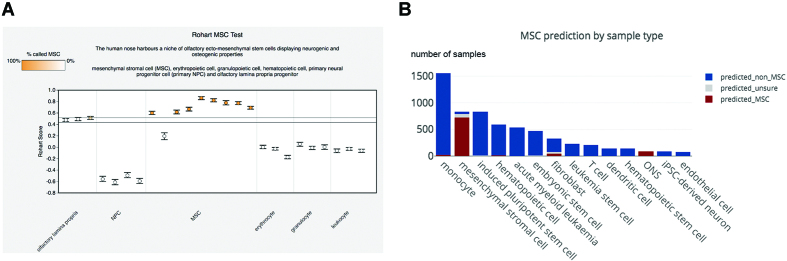
A screenshot of the Rohart MSC Test available for any human microarray dataset in Stemformatics. (**A**) Predicted Rohart score for each sample in the dataset, where scores above the prediction region indicate that samples are predicted as MSCs. (**B**) MSC predictions in Stemformatics for most common sample types. Not all sample types are shown.

An example of a collaborative focus within the Stemformatics portal is the classification of human mesenchymal stromal cells (MSC) using a custom-designed classifier, implemented as the Rohart MSC Test. This is an online implementation of a supervised method (15), which makes the prediction based on the signature of key genes based on an original training set of 635 curated samples profiled on 15 different microarray platforms. This classifier can be run on any human microarray dataset, and provides a score that allows users to evaluate the similarity of a sample to a gold standard set of curated MSC. The resulting graph plots the Rohart score for each sample in a dataset, and a cutoff line shown can be used to determine if a sample can be regarded as an MSC by the algorithm (Figure 6A). Since its launch in 2016, >12 000 samples have been evaluated by the Rohart MSC classifier on an expanded number of microarray platforms, and it remains a faithful predictor of MSC samples (Figure 6B).

## CONCLUSION

Stemformatics is an online gene expression data portal, hosting a large number of easy-to-use features that facilitate exploration of high quality and relevant data sets by the stem cell community. All processed data are available for easy download. Custom analysis results can be hosted for each dataset as standalone reports, which creates a flexible workflow.

In addition to standard functions such as gene expression profile plots, Stemformatics also has novel integrative data tools that leverage both the quality and the quantity of datasets contained. An example of such a tool is the Yugene Graph, which ranks samples from highest to lowest expression for a given gene. These integrative tools greatly enhance the value of the datasets included.

Stemformatics is also a collaborative platform that has supported a number of projects for their data processing, analyses and hosting. Stemformatics is a unique and powerful resource for a wide range of research communities worldwide.

## DATA AVAILABILITY

All data are freely available at stemformatics.org, and downloadable from each dataset page or via multi-dataset download page. There are direct links to raw data repositories such as ArrayExpress (8) or Gene Expression Omnibus (9) for published datasets. Almost all functions are available without a user registration, except 'My Gene Lists' (saving gene sets on the server), and running jobs which include 'Hierarchical Cluster' and 'Gene Expression Profile' (find genes based on user defined expression profile). Note that there is also an option to use a guest account with a single click, which enables all these functions without requiring a registration.

The Stemformatics is an Open Source project with a number of public repositories. The main code base is available in the BitBucket repository (https://bitbucket.org/stemformatics/s4m_pyramid), while the data processing pipelines are also in BitBucket (https://bitbucket.org/stemformatics/stemformatics_tools, https://bitbucket.org/uqokorn/s4m_modules). Other links to our repositories can be found on our website under “About Us” → “Our Code”.

## COMPUTATIONAL RESOURCES

Stemformatics is hosted on the NeCTAR Research Cloud, a collaborative Australian research platform supported by the National Collaborative Research Infrastructure Strategy (NCRIS) and the Queensland Cyber Infrastructure Foundation (QCIF). This research project was undertaken with the assistance of resources and services from the National Computational Infrastructure (NCI) which is supported by the Australian Government, as well as compute resources of the Australian Institute for Bioengineering and Nanotechnology at the University of Queensland.

## Supplementary Material

Supplementary Data
