# Supplementary material for: Stemformatics: visualize and download curated stem cell data
Source: Nucleic Acids Res. 2018 Nov 8;47(Database issue):D841–6. doi: 10.1093/nar/gky1064 (PMC6323943; doi:10.1093/nar/gky1064)
Supplement: Supplementary Data [file gky1064_supplemental_files.pdf]

Supplementary Data

Sample type table (Table 1 from the main text)

Stemformatics holds all relevant sample metadata for a dataset. To accommodate the diversity of these data, key-value relationships are used: eg. “tissue”=”brain”, or “sex”=”female”. However this flexibility also creates a drawback, in that data is unstructured and cross-dataset queries become difficult (eg. “tissue”=”Brain” in one dataset may not match up with “tissue_of_origin”=“brain” in another). As a part of the ongoing effort to add structure to this data, we have added “generic_sample_type” field to all samples, and this is the field used to create this table.

Note that all sample fields including generic_sample_type are available from the sample table downloaded from Stemformatics using multi-dataset download page (https://www.stemformatics.org/workbench/download_multiple_datasets > export selected sample metadata).

Datasets passing quality control by journal (Figure 3 from the main text)

Stemformatics uses an in-house project management system to track dataset processing and can log quality control issues with datasets as they are processed. To produce Figure 3 of the main text, as well as S1 below, we looked up the journal of publication for failed datasets using their pubmed id and the journal impact factors using google and scimagojr.com.

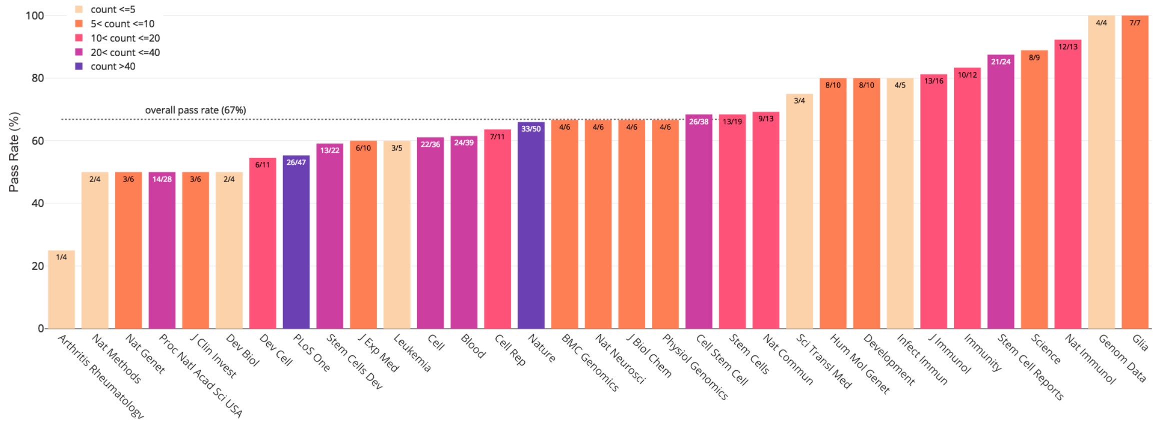

Figure Supplementary 1 (S1): Similar to Figure 3 of the main text, this plot shows the percentage of datasets passing quality control for journal of publication, but this time coloured by total numbers of datasets from the journal, rather than impact factor.

Gene correlation (Figure 4 from the main text)

The measure of correlation used here is the Pearson correlation. While a common measure of a linear relation between two variables, it can be sensitive to the sample size of the calculation. Additionally, the two platforms used here, Microarrays and RNA Sequencing, are based on physically different methods and have different noise profiles. Here we outline our method for keeping bias and errors within acceptable limits in order to produce Figure 4.

The procedure is:

1. Select datasets that have 20 or more samples. This leaves 92 Microarray datasets and 13 RNA Seq datasets.

2. Select gene-gene pairs that are measured in at least %50 of both Microarray and RNA Seq datasets.
3. Measure the correlation coefficient for each gene-gene pair in each dataset separately, then
4. Average the coefficients for each gene-gene pair across datasets in the respective platforms.

We keep this procedure simple and are interested in broadly whether any gene-gene pairs are consistently highly correlated, and whether our platforms can be compared to each other by their correlations. For RNA Seq datasets we work in TMM normalised (Robinson & Oshlack 2010) log2 CPM.

The motivations behind our choice of values for sample size cuts and expression thresholds are summarised in the panels of Figure S2:

Panel A: For a gene expression within an RNA Sequencing experiment in log CPM space, the scatter about the mean of a value increases as the mean itself decreases (e.g. Law et al 2014). Therefore, genes with lower average expression are noisier, and their correlations harder to reliably measure. Genes with low counts are often filtered out (for example as implemented in Love et al. 2014). We impose a lower expression threshold based how well we can recover correlations after we artificially introduce sampling noise. That is, for each gene, we resample it from the binomial distribution based on its mean value and the library sizes within the dataset. We perform this 200 times and correlate these resamples with each other - if the noise is small relative to the original variance, resamples should be well correlated (zero noise will produce correlations of 1). Otherwise noise will dominate, and low correlations will appear.

Panel A in S1 shows the mean-standard deviation plot (log2 space) for an example dataset. Each point is a gene. The points are coloured according to how well we recover their self-correlations after resampling as described above. Orange points have on average a resampled correlation > 0.7, while blue points have an average < 0.7. The value of 0.7 is often quoted as the threshold of biological significance (Allocco 2004), and the margin of ~0.3 in is line with findings in Panel C and D. Therefore, we use this as a guideline and implement a minimum threshold of -3.0 which removes the bulk of genes with very noisy data. This is performed for each dataset.

Panel B: Microarray platforms are treated in the same manner as per Rohart et al. 2016. They exclude genes below the Microarray detection threshold, based on the bi modality of the abundance distribution.

Only gene-gene pairs that pass these expression cuts in at least %50 of both Microarray and RNA Seq datasets analysed. This is to weed out pairs that are exclusive to one cell type, or are simply a chance appearance in only a small number of datasets.

In order restrict differences in correlation coefficients due to the different sample sizes in each dataset, we impose a minimum sample size threshold of 20. To assess effect of the differing sample sizes we subsampled datasets down to our minimum of 20 samples. We performed this 500 times for our 5 largest datasets and measured how each gene-gene pair coefficient differs after subsampling. The sample size of each dataset (6286, 6461, 7129, 7168, 7258) is respectively 384, 173, 191, 227 and 251. The results are shown in panels **Panel C** & **Panel D.**

Panel C: Shows results of repeatedly subsampling each dataset down to 20 samples. The y axis shows distribution of the *average difference* in correlation coefficients after

subsampling. Gene-gene pairs are binned by their original coefficient. Average differences are distributed relatively closely about 0.

Panel D: Similarly, to C, **Panel D** shows the distribution of the *standard deviation of the difference* in correlation coefficient for each gene-gene pairs after subsampling. Typical scatter due to the subsampling is ~0.2. Rarely does subsampling induce the scatter of 0.3 or above.

Subsampling differences for each gene pair are thus kept around are approximately ~0.2 and are unlikely to be driving the global trend observed in Figure 4.

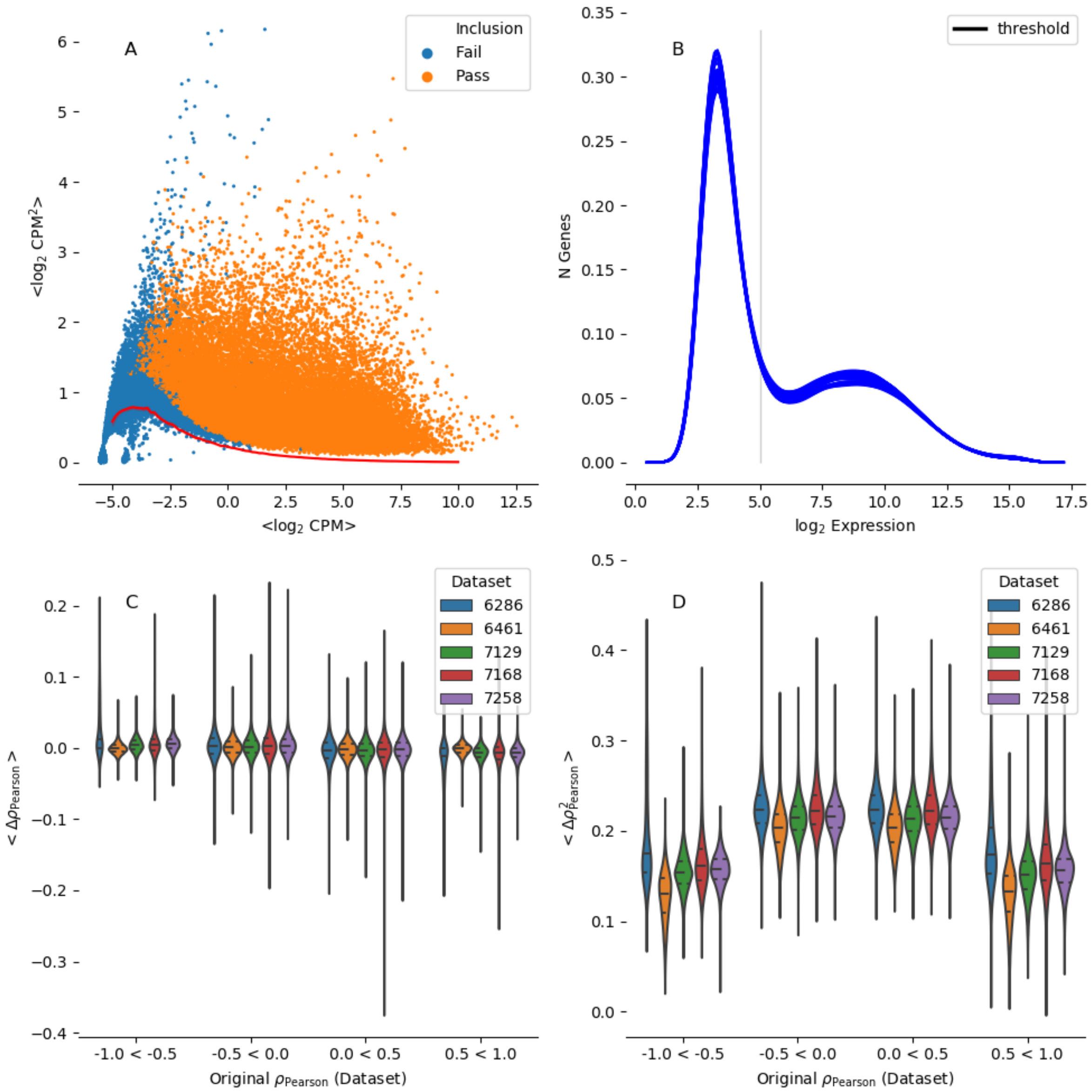

Figure Supplementary 2 (S2): Panel A shows an example mean - standard deviation (log2 space) relation within an RNA Seq dataset. Orange points are genes whose variance is well

above the noise, as estimated via resampling (see text), blue points are the remaining genes. Panel B: an example Microarray distribution showing bimodality. Panel C: the distribution of mean difference in correlation after subsampling five large datasets. Panel D: The distribution of standard deviations of difference in correlation after subsampling.

References

M. D. Robinson and A. Oshlack. (2010), A scaling normalization method for differential expression analysis of RNA-seq data. *Genome Biology*.

Law CW, Chen Y, Shi W, Smyth GK. (2014), Voom: precision weights unlock linear model analysis tools for RNA-seq read counts. *Genome Biology.*

Love MI, Huber W, Anders S. (2014) Moderated estimation of fold change and dispersion for RNA-seq data with DESeq2. *Genome Biology.*

Rohart F, Mason EA, Matigian N, Mosbergen R, Korn O, Chen T, et al. (2016) A molecular classification of human mesenchymal stromal cells. *PeerJ*.

Allocco DJ, Kohane IS, Butte AJ. (2004) Quantifying the relationship between co-expression, co-regulation and gene function. *BMC Bioinformatics*.
